# Interrogating the Construct of PRETCO-Oral: Longitudinal Evidence From Raters and Test-Takers

**DOI:** 10.3389/fpsyg.2022.896453

**Published:** 2022-07-12

**Authors:** Zhiqiang Yang, Yongqiang Zeng, Zhifang Li, Zhiqing Lin

**Affiliations:** ^1^School of Foreign Studies, Chongqing University of Science and Technology, Chongqing, China; ^2^Center for Linguistics and Applied Linguistics, Guangdong University of Foreign Studies, Guangzhou, China; ^3^Guangdong Teachers College of Foreign Language and Arts, Guangzhou, China; ^4^Department of Foreign Language Teaching of Basic Medicine School, Army Medical University, Chongqing, China; ^5^School of English Studies, Shanghai International Studies University, Shanghai, China

**Keywords:** PRETCO-Oral, construct validity, speaking assessment, longitudinal reliability, measurement invariance

## Abstract

In speaking assessment, many factors such as characteristics of test-takers, test tasks, rating bias, etc. may affect the speaking performance of test-takers. Besides, the stability of raters' rating of a speaking test might pose a threat to its reliability, validity, and fairness, which calls for longitudinal construct validation of the speaking test. This study explores the construct validity of PRETCO-Oral through analysis of data retrieved from various sources, including longitudinal ratings of performances of test-takers across four occasions, and perceptions of the construct of PRETCO-Oral from both raters and test-takers. The results indicate that raters' ratings keep stable and the PRETCO-Oral assessment is equipped with longitudinal reliability; tasks of Interpretation and Presentation represent a large amount of variance of the construct, while those of Reading Aloud and Question and Answer seem to be construct-underrepresented, as evidenced via analyzing the data collected from perceptions of raters and test-takers upon the test construct. Finally, factors that threaten the construct representation are also discussed.

## Introduction

Over the decades, the central enterprise in language testing has been the study of validity (Fulcher and Davidson, 2007) where construct validity is a key concept underpinning the relationship between the test and the proposed interpretations (Cronbach and Meehl, 1955; Messick, 1989; Bachman and Palmer, 1996; Kim and Crossley, 2020). The ability to speak in a foreign language is at the center of what it means to be able to use a foreign language, yet speaking is the most challenging skill to assess reliably and many factors such as characteristics of test-takers, test tasks, rating bias, etc. may affect test-takers' speaking performance (Alderson and Bachman, 2004). In addition, the stability of raters' rating of a speaking test might pose a threat to its reliability, validity, and fairness (Yang et al., 2021), which calls for longitudinal construct validation of the speaking test. Most of the extant literature of interest, however, centers on a synchronic study of construct validity (Sawaki, 2007; Sawaki et al., 2009; Fulcher, 2015; Cai, 2020), and few of them collect evidence from the perspective of test-takers whose perceptions or attitudes might be a vital source of evidence for construct validity (Fan and Ji, 2014). To fill the gap, this study sets out to look into the construct of the Practical English Test for College Oral (PRETCO-Oral for short henceforth) longitudinally from the perspectives of both raters and test-takers.

### The Genesis of PRETCO-Oral

Since the founding of the People's Republic of China, China's higher education is considered to comprise three layers, i.e. Postgraduate, Undergraduate, and Vocational education. There are 1,468 vocational colleges in China by 2020, taking up 53.6% of all colleges in China. Vocational colleges constitute a crucial part of China's higher education, albeit it ranks lowest among the three levels. To advance its development, English teaching is incorporated into the objective of vocational college education in a bid to develop students' practical English ability (Liu et al., 2010), and the English Teaching Requirement for Vocational Colleges (trial) was enacted and administered in 2000. The Practical English Test for College (PRETCO for short henceforth) was launched in such a context with the purpose of examining whether the English proficiency of vocational college students meets the requirement stipulated in the English teaching syllabus and whether it satisfies the demand of social and economic development upon vocational college students.

PRETCO is administered under the auspice of the Practical English Test Committee empowered by the Education Ministry of China and is composed of two separate sub-tests, i.e., paper-and-pencil PRETCO and PRETCO-Oral, which cover all four practical skills (Liu et al., 2010). The paper-and-pencil PRETCO test battery consists of two parts, i.e., Band A (PRETCO-A), and Band B (PRETCO-B), implemented twice a year, which, to a large extent, is a large-scale and high-stakes test, as in some vocational colleges to pass the PRETCO-B is a prerequisite for graduation (Shen, 2014). The number of examinees sitting for the written PRETCO has reached 4,000,000, and it keeps rising (Liu et al., 2010). Regarding the PRETCO-Oral, it is a computer-mediated test administered a month before the paper-and-pencil PRETCO. Sitting for the PRETCO-Oral is of test-takers' free will, and it is said that a certificate of PRETCO-Oral might help increase students' competitive edge in future employment.

The PRETCO-Oral lasts about 20 min in the form of man-computer dialogue. For each task, 1 to 1.5 min' preparation is allowed for test-takers, and two to four parallel test sheets will appear on each test occasion. PRETCO includes four tasks, which seem to be devised involving three ways of construct definition, namely, Reading aloud, designed to examine test-takers' speaking ability, consisting of intonation, pronunciation, and fluency; Question and Answer task, a semi-direct test with three “Questions” and three “Answers” aiming at measuring examinees' interactional and communicative skill; Chinese-English Interpretation, resembling the task in the workplace, and Presentation task where figures or tables about a company or its production and so forth are provided and test-takers are required to describe and comment on those charts.

The four tasks are rated separately according to four respective 7-band rating scales, ranging from 0 to 4 points (0, 1, 2, 2.5, 3, 3.5, 4), with a total score of 16 points (4 points × 4 tasks). Before rating, all the raters are trained to be familiar with the rating criteria, and the typical response of test-takers on each rating category, for the purpose of ensuring the scoring consistency. Each examinee's performance is scored by two different raters to secure reliability. As stipulated in the PRETCO-Oral syllabi, the examinees' performance will be categorized into three types on the basis of the rating results: Excellent, Pass, and Not Pass. Nearly two decades' operation notwithstanding, the validation of the PRETCO-Oral construct was not yet touched, and this study is going to fill the niche.

### Literature Review

In speaking assessment, rating scale and the way it is interpreted by raters represent the de-facto test construct (Knoch, 2009; Fulcher, 2015), studies on the rating scale and especially the quality of raters' ratings are of key concern among researchers of speaking language assessment. As speaking assessment entails subjective ratings of human beings which might give rise to rating bias or rater effect (Kim, 2015), reliability investigation employing many-facets Rasch measurement (MFRM in brief) gains increasing popularity to fathom out the extent to which raters' ratings or test scores are consistent and valid (Lumley and McNamara, 1995; Upshur and Turner, 1999; Bonk and Ockey, 2003; Eckes, 2005; Yang, 2010; Kang et al., 2019). It should be pointed out that rater effects are dynamic and will change over time (Myford and Wolfe, 2004) and the stability of raters' ratings might pose a threat to their rating quality and hence undermine test validity (Zhao et al., 2019), which highlights the need to conduct a longitudinal study regarding raters' rating reliability.

The pity is that quite a few studies are of this type with the exceptions of Lumley and McNamara (1995), Yang (2010), and Bonk and Ockey (2003), whose findings, however, were at odds with each other. Some claim that raters demonstrate different degrees of changes across three occasions of ratings (Lumley and McNamara, 1995), and others maintain that there was a huge discrepancy concerning raters' severity, which was not stable over time for individual raters (Bonk and Ockey, 2003). The instability of raters' rating was also detected in the study of Yang (2010) who, nonetheless, pointed out that it made no difference to the overall rating quality of raters. Therefore, more literature on longitudinal studies is needed in the field of speaking assessment.

The desirable reliability of raters' rating guarantees valid scores for speaking tasks. Construct validation lies in collecting evidence that test scores manifest the underlying construct that the test intends to assess (Kim and Crossley, 2020), which could, traditionally, be accomplished by means of examining the internal structure of the speaking test using confirmatory factor analysis (CFA) (Sawaki, 2007; Sawaki et al., 2009; Fan and Bond, 2016; Cai, 2020). Cai (2020) attempted to unveil the relationship between language ability and topical knowledge using CFA conducted on the scores of Test for English Majors, Band 4, Oral test (TEM4-Oral), and the results asserted that oral task performance is a multifaceted construct that includes both language ability and topical knowledge. Another case in point was done by Sawaki's (2007) in which CFA was used to assess the goodness of fit of CFA models that explain the structural relationships between the five rating scales of role-playing speaking tasks in a Spanish speaking assessment, which substantiates the existence of a single underlying dimension. Similarly, Sawaki et al. (2009) conducted CFA to examine the factor structure of TOEFL iBT, and the result indicated that the TOEFL iBT's integrated Speaking and Writing problems could be primarily referred to as assessments of speaking or writing skills, respectively. Although these studies present substantial support for the goodness of fit of CFA models, their findings might not hold across different samples, which necessitates longitudinal or cross-validation to examine whether the model estimates are stable across different occasions (Xi, 2010). On top of that, a mere quantitative method might trigger a specific method effect, and triangulation of methods are recommended (Long, 2005; Xie, 2010), such as interview adept at delving into raters' thought about their usage of rating scales, or questionnaire for collecting test-takers' perceptions upon the construct of speaking assessment.

Aside from the rater stability, test takers' perceptions or attitudes should be deemed as a vital source of evidence for construct validity (Messick, 1989; Fan and Ji, 2014). For instance, test takers' perceptions might impact their performance on test tasks (Cheng, 2005), and extant studies show that both test candidates for high-stakes tests, such as TOEIC (Zhou and Yoshitomi, 2019) or IELTS (Rasti, 2009) and those of low-stakes school-based English test (Fan and Ji, 2014) react positively toward the construct of those tests mentioned. Furthermore, test takers' perceptions of assessment affect the measurement of the intended construct (Xie, 2011). Notwithstanding the crucial role of test takers' perception, studies about their perceptions toward rating scales or the construct of speaking assessment in particular, however, are scarce. This study will investigate the construct of a speaking test, namely, PRETCO-Oral, from test-takers perceptions on the rating scales or the construct of this test.

In a word, most of the existing literature of interest centers on the synchronic study of construct validation of speaking assessments, and few of them collect evidence from the perspective of raters and test-takers simultaneously. To fill that niche, this study will look into the construct of PRETCO-Oral longitudinally based on triangulation of data derived from test scores, questionnaire of test takers' perception, and raters' interview, and endeavor to answer the following questions:

**Question 1**: Is the scoring of raters in PRETCO-Oral longitudinally reliable?
**Question 2**: To what extent can the construct of PRETCO-Oral be interpreted based on test scores?
**Question 3**: Does the construct of PRETCO-Oral keep the same manner longitudinally?
**Question 4**: To what extent can the construct of PRETCO-Oral be interpreted from the perspective of PRETCO-Oral test-takers?

## Methods

### Participants

To investigate the reliability of PRETCO-Oral longitudinally, 5,032 test-takers of four consecutive occasions of PRETCO-Oral with respective 1,356 (20 raters), 1,351(20 raters), 870 (12 raters), and 1,455 (20 raters) were involved in this study. The exam classroom can accommodate a maximum of 120 test-takers at one time. Thus, test-takers, categorized into 8–13 cohorts, sat for the test successively for each occasion. On the fourth occasion, 464 examinees with 97 males (21%) and 366 females (79%) from nine universities and vocational colleges were willing to participate in the questionnaire investigation aimed at gleaning information about test-takers' perception of the construct of PRETCO-Oral. These test-takers answered the questionnaire at www.wenjuan.com on the computers in the neighboring teaching room immediately after they finished the PRETCO-Oral. Given that the majority of examinees did not take the test before, there was a routine operation of test training of roughly 20 min for each cohort of examinees. A total of 12–20 raters for each occasion were invited from about 10 different universities or vocational colleges for the scoring of PRETOC-Oral. The raters except one or two new ones were experienced raters for more than 3 times of rating, yet all of them would receive an almost half-day rater training before the rating. After the rating of PRETCO-Oral, 8 raters, also college English teachers, of four males and four females with at least 6 times of PRETCO-Oral rating experience received interviews about their perspectives on the four tasks and construct of the test. Among the rater interviewees are two English major teachers (Hanna and Lily) and six non-English major teachers comprising one from vocational institute (Lucas) and five from general college (Eric, Shelly, Chris, Chad, and Dora). All names in the bracket are pseudonyms.

### Instrument

The instruments in this study include the test PRETCO-Oral itself, a questionnaire, and an interview. The questionnaire was designed with reference to the rating criteria and test syllabus of PRETCO-Oral. Taking the task of “Presentation” for example, its rating scale is depicted as “Can present the important information contained in the picture clearly and coherently with comments, and the expression conforms to the language norm”; The requirements for this task stipulated in the syllabus include “The examinee is asked to present a coherent statement according to the content of the figures or tables with prompts and express his/her personal opinions or comments.” Those descriptors mentioned are decomposed as “I can choose the right words when I make the presentation; I can express myself in correct sentences; I can describe all the charts properly; I can organize the appropriate language make a comment; I can focus on the coherence of my presentation” in the questionnaire. Included in the questionnaire are four sections concerning Personal information, Examinees' Knowledge about PRETCO-Oral, Perception about the Construct of PRETCO-Oral (see Appendix B in the Supplementary Materials), and Impact of PRETCO-Oral. The interview adopted in this study is a semi-structured one with key questions like “What does the PRETCO-Oral test in general?”, “What does the task of Reading aloud test?”, “What does the task of Question and Answer test?”, “What does the task of Interpretation test?” and “What does the task of Presentation test?”. Both the questions and answers were carried out in Chinese.

### Data Collection

Examinees' performances were recorded and their names and ID numbers were concealed when delivered to each rater at random. Each examinee's performance was rated by two different raters, thus generating 10,064 scores in total (5032 × 2) on four occasions. The questionnaire was delivered through www.wenjuan.com and 464 test-takers' answering was collected without missing data. A small number of examinees, however, answered the questionnaire much faster, which may affect the quality of the data. This study thereby excludes those questionnaires with 20 or more consecutive same options. Eventually, the number of valid questionnaires was 392 (84.5%) with 68 boys (17.3%) and 324 girls (82.7%), which is of high reliability with a value of 0.935 of the Cronbach's α. For the eight raters' interviews, the average time for each rater lasts 20.5 min, totaling 164 min, which were transcribed verbatim, yielding 43,598 Chinese characters.

### Data Analysis

During rater, training raters are required to follow the four rating scales of 7 levels while assigning scores, and meanwhile, for the sake of the convenience of Rasch statistics, all of the original rating scores (0, 1, 2, 2.5, 3, 3.5, 4) were transformed to seven degrees (1, 2, 3, 4, 5, 6, 7). To answer the first research question, the multi-facet Rasch model was applied to examine whether the rater's rating of examinees' performance is reliable and stable. The rating scale of PRETCO-Oral differs from one task to another. As a result, the model used in the analysis was a four-facet partial credit model (Linacre, 2002b), including examinee ability, rater severity, test sheet, and task difficulty. Data analysis of rating was performed on FACETS 3.71.3 (Linacre, 2013). Given that the PRETCO-Oral involves parallel test sheets and a large number in the dataset were randomly assigned to sheets, it is safe to posit that the mean ability values of examinees assigned to different sheets were equal (Bonk and Ockey, 2003). We anchored the mean value of examinees on different sheets at zero logits, and subset connectedness was achieved.

**Figure 1:**
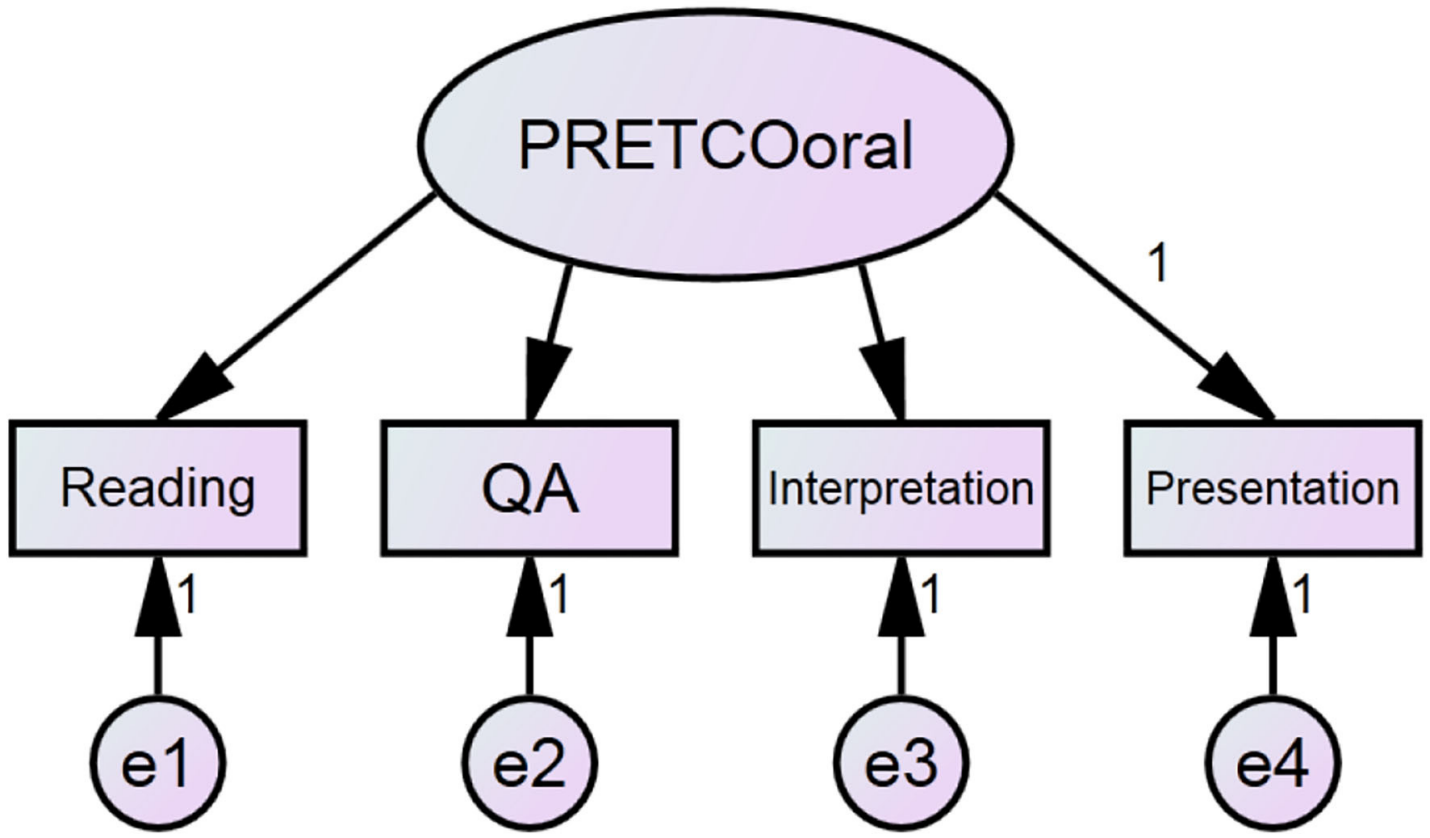
Hypothetical model 1 of PRETCO-Oral. Reading and QA refer to tasks Reading Aloud and Questions and Answer, similarly hereinafter.

To answer the second research question, interpretation of the construct could be realized through CFA (Cai, 2020), where the original average score of two raters will be used. Considering that there are four tasks of PRETCO-Oral, hypothetical Model 1 regarded performance on the four tasks as a single construct, as is shown in Figure 1.

In addition, the qualitative method was also used to investigate the construct of the oral test further by interviewing the rater. Interview data of 43,598 Chinese characters were imported into NVivo 11 for analysis and the data were coded following an iterative inductive coding paradigm (Saldaña, 2013) on grounds of the PRETCO-Oral and its four tasks. For the purpose of improving the reliability of coding, data were coded again one month later by the same coder (Cohen's kappa = 0.89). The results consist of 233 references and 10 nodes covering the topics of the overall construct of PRETCO-Oral (30 references), Reading Aloud (25 references), Question and Answer (32 references), Interpretation (38 references), Presentation (27 references), factor affecting the construct (27 references), the overall impression over the stability of PRETCO-Oral (17 references), construct irrelevant variables (16 references), the familiarity of the four tasks (15 references), and discrepancy of viewpoints upon rating scale (6 references).

**Figure 2:**
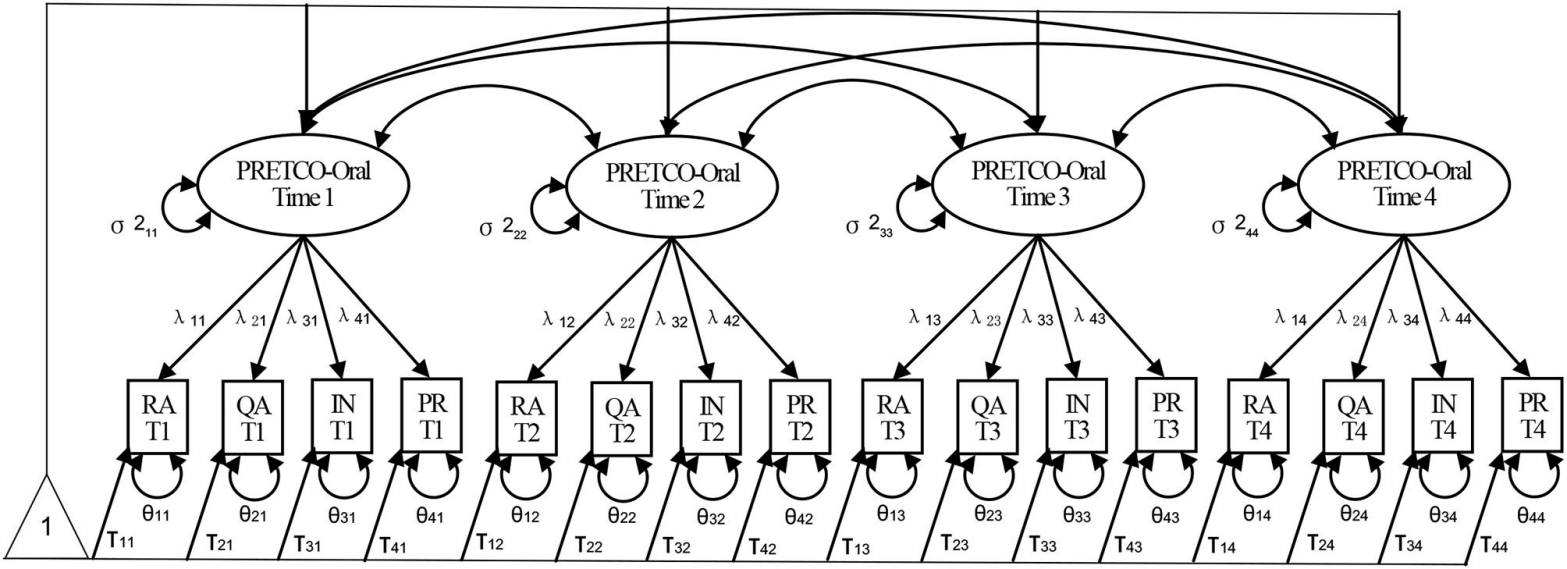
Longitudinal confirmatory factor model for PRETCO-Oral. σ^2^, λ, τ, and θ refer to variance, factor loading, intercept, and unique factor variance (Widaman et al., 2010).

To answer the third question, the separate analysis of PRETCO-Oral test scores of the four occasions were examined and compared. Furthermore, multi-group CFA (MG-CFA) was employed to examine whether the PRETCO-Oral maintains measurement invariance across the four occasions of assessments. The following steps were taken (see Figure 2): (1) configural invariance, i.e. testing whether the constructs have the same pattern across times; (2) metric invariance (weak factorial), i.e. examining the equivalence of factor loadings through constraining corresponding first loadings to be invariant across time; (3) scalar invariance (strong factorial), i.e., testing the equivalence of item intercepts via constraining corresponding first intercepts to be invariant across time; and (4) residual invariance (strict or invariant uniqueness), referring to the equivalence of item residuals or unique variances (Widaman et al., 2010; Putnick and Bornstein, 2016).

**Figure 3:**
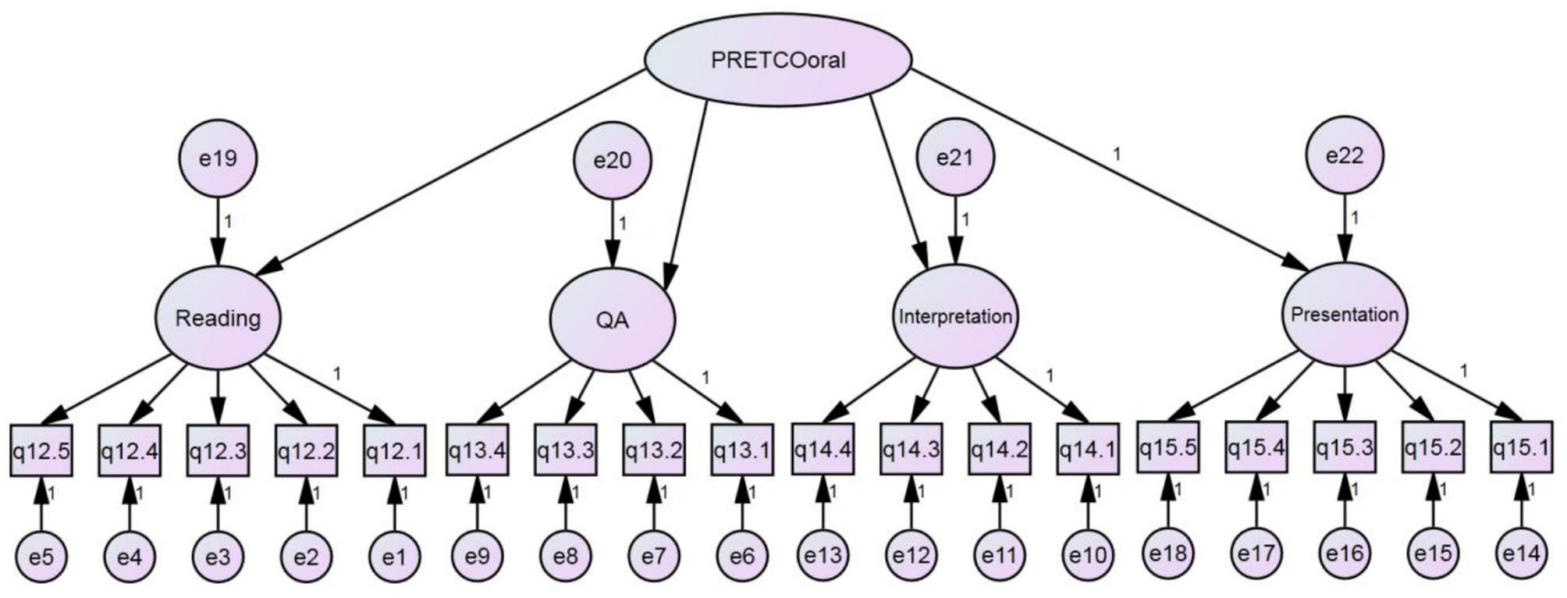
Hypothetical model 2 of PRETCO-oral.

To answer the fourth research question, CFA was also conducted based on the basis of the questionnaire data. In line with model 1, hypothetical model 2, illustrated in Figure 3, considered the second-order potential factor as the construct of PRETCO-Oral from the perspective of test-takers. CFA was accomplished by AMOS 21.0.

## Results

### Rasch Analysis

The important indicator of internal consistency of rating reliability is Infit Mean-Square (Infit in brief) (Linacre, 2002b, 2013; Eckes, 2005). There are two versions of Infit range: the broad range (0.5–1.5) and the narrow range (0.7–1.3) (Eckes, 2005). With regard to the former, rating data from raters with Infit value greater than 1.5 have a great chance to misfit the model, while those of raters with Infit value less than 0.5 tend to overfit the model (Linacre, 2002b). Infits of all raters, except R5 (1.68) on the fourth occasion, fell in the range of 0.5 to 1.5, which suggests that the raters' ratings were generally consistent from the broad range perspective longitudinally (see Table 1).

**Table 1:** Rater mean-square infit statistics.

**1 st Occasion**		**2 nd Occasion**		**3 rd Occasion**		**4 th occasion**	
**Raters**	**Infit**	**Raters**	**Infit**	**Raters**	**Infit**	**Raters**	**Infit**
R17	1.48	R5	1.29	R1	1.21	R5	1.68
R5	1.29	R19	1.28	R10	1.18	R3	1.25
……	……	……	……	……	……	……	……
R6	1.03	R16	0.99	R9	1.06	R13	1.01
……	……	……	……	……	……		
R16	0.77	R20	0.74	R2	0.81	R14	0.78
R12	0.63	R18	0.55	R4	0.69	R2	0.76

*On different occasions of rating, every rater was renumbered, therefore Rater 5 in the 2^nd^ occasion is different from Rater 5 on the 4^th^ occasion*.

With reference to the latter, viz. narrow range of Infit, ratings of rater R17 (1.48 > 1.3) and Rater 5 (1.68 > 1.3) from the first and fourth occasions tended to misfit the model, and those of raters R12(0.63), R18(0.55), and R4(0.69), less than 0.7, tended to overfit the model, which might present a central tendency during rating (Myford and Wolfe, 2004). Generally, the narrow range of infit is reasonable for “high stakes” tests (Wright, 1996), and the broad range of infit is productive for measurement (Linacre, 2013).

The separation of examinees or raters is an indicator of the spread of examinee performance or the rater's severity in comparison with their precision. The examinee separation ratio of 2.72 in Table 2 suggests that the spread of the examinee performance measures is more than two times larger than the precision of those measures (Myford and Wolfe, 2004). When the separation reliability is less than 0.5, the differences between the measures are primarily owing to measurement error (Fisher, 1992; Myford and Wolfe, 2003). The high degree of examinee separation reliability on four occasions (0.88, 0.89, 0.91) larger than 0.7 indicates raters were able to discriminate between the examinees with high reliability, and rater separation reliability of 0.98, 0.99, 0.96, and 0.98 implied that raters were significantly different in terms of their severity (Myford and Wolfe, 2004). Table 2 shows raters longitudinally maintain a high degree of intra-rater consistency in differentiating examinees' performance while exhibiting significant discrepancy between severe and lenient raters or poor inter-rating consistency.

**Table 2:** Measurement of examinees and raters facets.

**Facets**	**Occasions**	**Separation**	**Reliability**	**Chi-sq**.	**d.f**.	***p*** **value**
Examinees	First	2.72	0.88	9,638.7	1,355	0.00
	Second	2.79	0.89	9,720.9	1,350	0.00
	Third	3.28	0.91	8,484.0	869	0.00
	Fourth	2.71	0.88	9,641.6	1,454	0.00
Raters	First	7.74	0.98	1,285.4	19	0.00
	Second	10.62	0.99	1,962.9	19	0.00
	Third	4.81	0.96	386.7	11	0.00
	Fourth	8.05	0.98	1,330.2	19	0.00

Table 3 shows the longitudinal usage of each rating category of the four tasks. Three scale categories, namely 2.5, 3, and 3.5 points, are overused by all the raters for each PRETCO-Oral occasion, taking up nearly 90% of all the ratings, which indicates that raters tend to exhibit a central tendency effect as a consequence of their inability to accurately assess examinees of extremely high or low proficiency, or their poor understanding of the rating scale. The central tendency of raters' rating on the task of Reading Aloud might probably be due to its poor quality of rating scale. In addition, the 4 point category of the last two tasks was seldom used, and the task of Presentation in particular, no observation was found in this category diachronically.

**Table 3:** Longitudinal usage of each category of the four tasks (%).

**Tasks**	**Occasions**	**0** **point**	**1 point**	**2** **points**	**2.5 points**	**3** **points**	**3.5 points**	**4** **points**	**Total %**
Reading aloud	First	0	1	7	23	44	22	3	100
	Second	0	1	7	23	45	21	3	100
	Third	1	1	6	25	45	19	3	100
	Fourth	1	2	13	24	36	20	4	100
Question and answer	First	12	21	24	22	14	6	1	100
	Second	13	20	24	22	14	6	1	100
	Third	11	21	22	19	17	8	2	100
	Fourth	20	22	22	17	12	6	1	100
Interpretation	First	2	10	27	30	24	6	1	100
	Second	2	9	26	34	25	4	0	100
	Third	5	12	30	26	21	6	0	100
	Fourth	3	12	21	24	27	12	1	100
Presentation	First	3	13	38	23	19	4	0	100
	Second	2	9	28	31	25	5	0	100
	Third	5	13	35	25	18	4	0	100
	Fourth	3	11	28	26	24	8	0	100

### CFA Based on PRETCO-Oral Scores

The fit statistics of four events of PRETCO-Oral were calculated using CFA, where a series of measures, including CMIN/X^2^, Root Mean Square Error of Approximation (RMSEA), Goodness of Fit Index (GFI), Adjusted Goodness of Fit Index (AGFI), Normed Fit Index (NFI), Tucker Lewis Index (TLI), and Comparative Fit Index (CFI), etc., were developed to evaluate the model fit (Hair et al., 2014). Because the X^2^ statistics are less meaningful as sample sizes become large, this study will not refer to this index.

Though an absolute cutoff value of RMSEA is disputed, it is well accepted that values less than 0.05 means good fit, those as high as 0.08 indicate “reasonable errors of approximation in the population” (p:80), and values greater than 0.10 imply poor fit (Byrne, 2010). The AGFI, one of the parsimony fit indices, is lower than GFI values in relation to model complexity, whose value of greater than 0.90 suggests a good fit (Byrne, 2010; Hair et al., 2014). The rest indexes' values of greater than 0.95 were considered good since there are only four observed variables in the hypothetical model 1 of PRETCO-Oral (Hair et al., 2014). For the large sample, values of indices, taking TLI for example, close to 0.95 are indicative of a good fit (Byrne, 2010).

As is seen in Table 4, the results of the CFA revealed that model 1 of PRETCO-Oral based on scores of four tasks demonstrated a longitudinally good fit except for the second occasion where the RMSEA was 0.131, larger than 0.08.

**Table 4:** Fit statistics for four occasions of hypothetical model 1 of PRETCO-oral.

**Occasions**	**CMIN/X^2^**	**df**	**GFI**	**AGFI**	**NFI**	**CFI**	**TLI**	**RMSEA**
			**>0.95**	**>0.90**	**>0.95**	**>0.95**	**>0.95**	**<0.08**
First	7.352	2	0.997	0.987	0.996	0.997	0.991	0.044
Second	48.264	2	0.983	0.914	0.980	0.981	0.942	0.131
Third	5.299	2	0.997	0.985	0.997	0.998	0.995	0.044
Fourth	12.016	2	0.996	0.980	0.996	0.997	0.990	0.059

What's more, the size of factor loading should be considered as key evidence for construct validity when using CFA. Standardized factor loadings exceeding 0.50 could be accepted. Ideally, and 0.7 or higher loading estimate is significant, the square of which equals around 0.5, “explaining half of the variance in the item with the other half being error variance” (p:618) (Hair et al., 2014).

**Figure 4:**
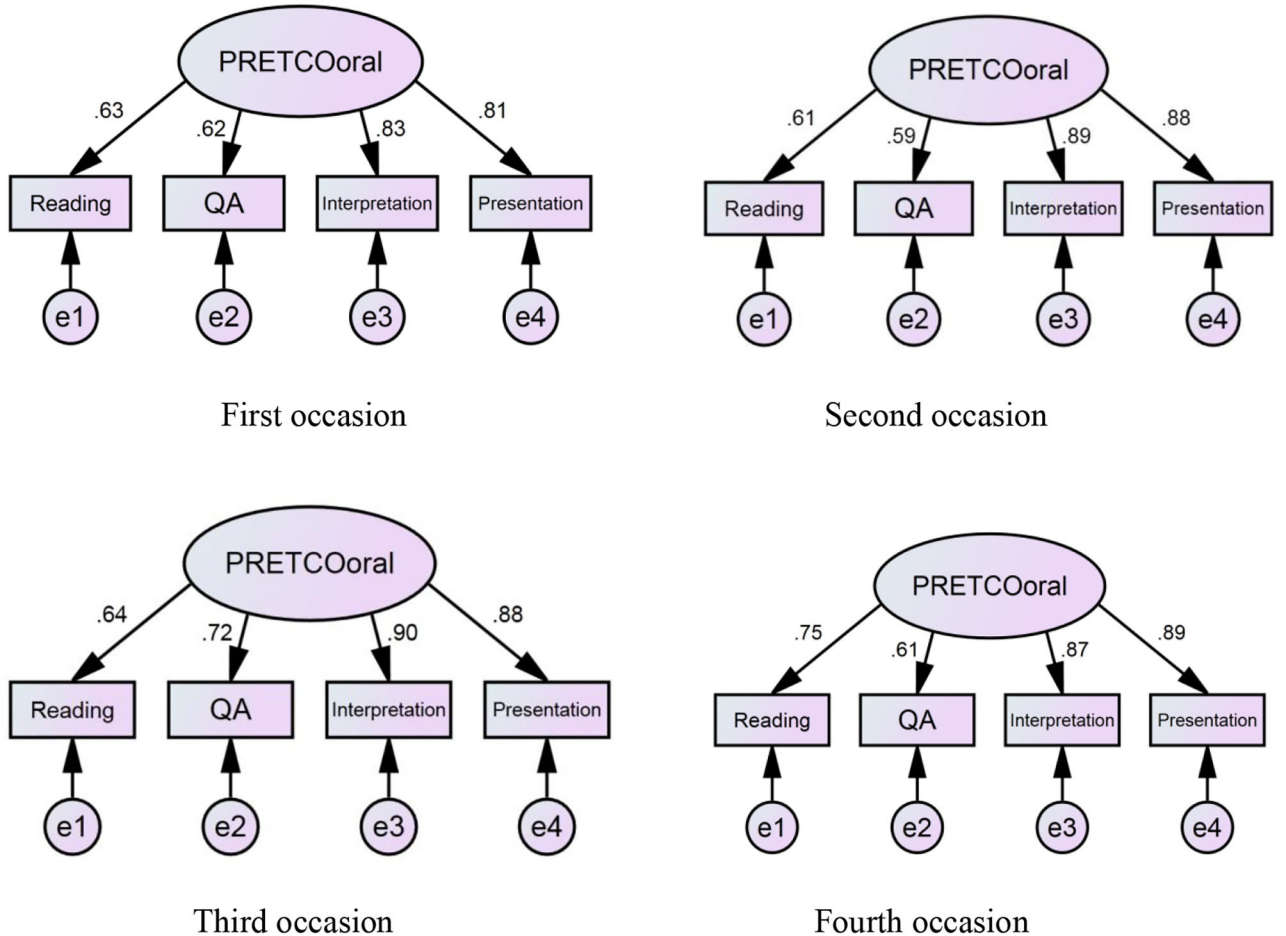
Four occasions of model 1 of PRETCO-oral.

It can be seen from Figure 4 that the factor loadings of the four tasks range from 0.59 to 0.90 over four periods of PRETCO-Oral tests, and those of the first two tasks exhibit much lower values on average (0.66, 0.64) than those of the Interpretation and Presentation tasks (0.87, 0.87) on a longitudinal basis. Taking Question and Answer, for example, the average loading factor only explains 40.9% (0.64 × 0.64) variance in the task with 59.1% of the rest being error variance. In contrast, factors of Interpretation and Presentation accounted for 75.7% (0.87 × 0.87) variance on average with 24.3% left being error variance.

To examine whether the measurement of PRETCO-Oral keeps equitable across the four-time points of assessment, changes in CFI (ΔCFI) can serve as the indicator when measurement invariance constraints are added. ΔCFI with the value smaller than or equal to −0.01 indicates that measurement invariance of test instrument should hold (Cheung and Rensvold, 2002). It can be seen from Table 5, the model of configural invariance has overall goodness-of-fit indices which suggests that this model fits the data well (RMSEA = 0.040 < 0.08; CFI = 0.993 > 0.9; TLI = 0.978 > 0.9) and that the pattern of loadings of the four tasks of PRETCO-Oral keeps equitable across the four sessions of assessments (Putnick and Bornstein, 2016).

**Table 5:** Measurement invariance of PRETCO-oral.

**Model**	**CMIN**	**df**	**RMSEA**	**CFI**	**TLI**	**ΔCFI**
configural	72.928	8	0.040	0.993	0.978	
metric	125.562	17	0.036	0.988	0.983	−0.005
scalar	595.744	29	0.062	0.936	0.947	−0.048
residual	799.285	41	0.061	0.915	0.950	/

Since the configure invariance is supported, the test of metric invariance or weak invariance can be achieved by comparing the metric model with constrained factor loadings to the configural model. It turns out that the constrained model is of acceptable overall model fit (RMSEA = 0.036 < 0.08; CFI = 0.988 > 0.9; TLI = 0.983 > 0.9), and the size of the factor loadings of PRETCO-Oral are the same across the four times of assessments (ΔCFI = −0.005). In a similar vein, the test of scalar invariance is conducted by constraining the item intercepts to be equivalent. Notwithstanding an acceptable overall model fit (RMSEA = 0.062 < 0.08; CFI = 0.936 > 0.9; TLI = 0.947 > 0.9), a comparison of scalar model to metric model demonstrates scalar non-invariance (ΔCFI = −0.048), which means that at least one item intercept differs across the four sessions of PRETCO-Oral assessment.

### CFA Based on Questionnaire Data

**Figure 5:**
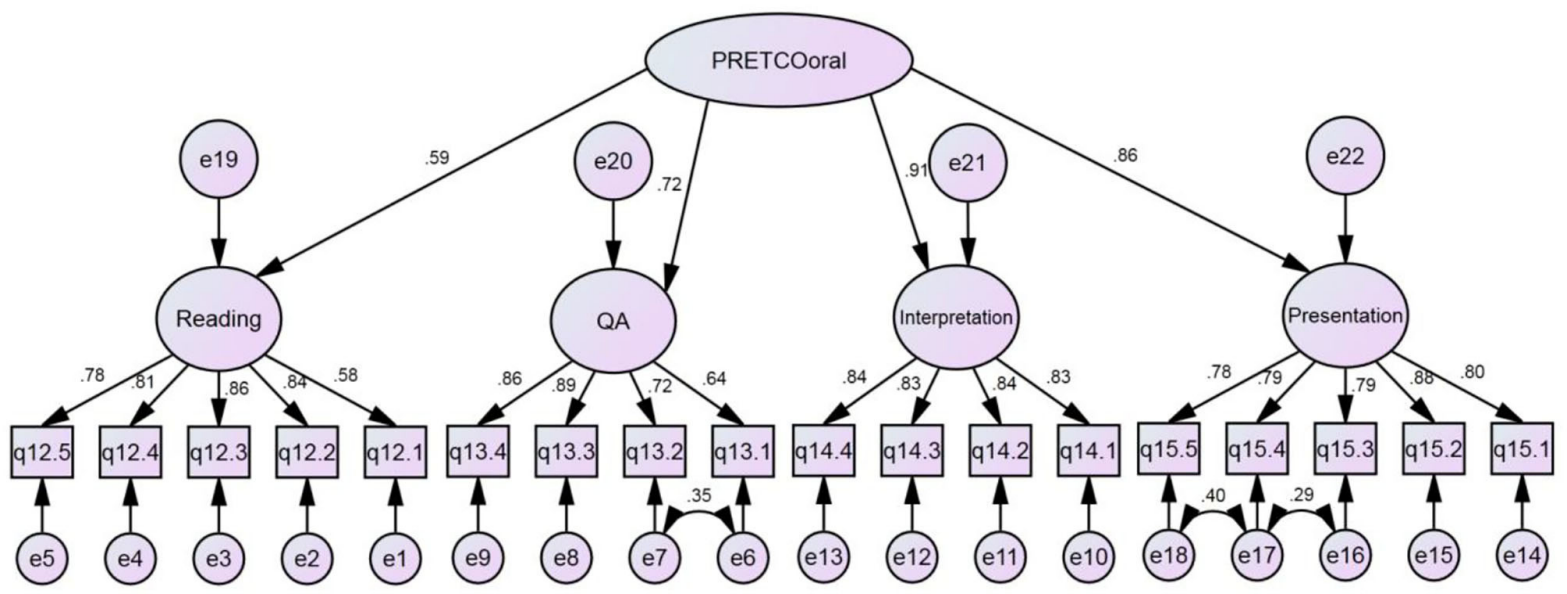
Hypothetical model 2 of PRETCO-oral based on questionnaire data.

The fit statistics of hypothetical model 2 of PRETCO-Oral were carried out by questionnaire data. As the sample of questionnaire data involving 18 observed variables is larger than 250, indexes such as GFI, NFI, CFI, and TLI with values greater than 0.92 indicate a good fit (Hair et al., 2014). Table 6 presents the goodness-of-fit indices for the hypothetical model 2, suggesting that the fit of the model was not satisfactory (GFI = 0.901 < 0.92; AGFI = 0.870 < 0.90) and accordingly modification indexes are requested (Byrne, 2001). The model fitted the data well, as is seen in Table 6 after the model was slightly modified. Loading factors of the four tasks were 0.59 (Reading Aloud), 0.72 (Question and Answer), 0.90 (Interpretation), and 0.86 (Presentation) presented in Figure 5, sharing many similarities with those gained in Model 1 on the basis of PRETCO-Oral scores.

**Table 6:** Fit statistics of hypothetical model 2 of PRETCO-oral.

	**CMIN**	**df**	**GFI**	**AGFI**	**NFI**	**CFI**	**TLI**	**RMSEA**
	/	/	>0.92	>0.90	>0.92	>0.92	>0.92	<0.08
Before M	366.958	131	0.901	0.870	0.927	0.952	0.944	0.068
After M	256.242	128	0.932	0.909	0.949	0.974	0.969	0.051

“*Before/After M” stands for before/after modification of the model*.

The statistics aforementioned can serve as evidence that the four tasks could well represent the latent variable namely the construct of PRETCO-Oral either based on the scores or from the perspective of test-takers. However, the two types of data seem to point toward the same conclusion that the Reading Aloud task is not adequate in explaining the construct of PRETCO-Oral, followed closely by the task of Question and Answer.

## Discussion

**RQ 1**: Is the scoring of raters in PRETCO-Oral longitudinally reliable?

The results of the Infit statistics of raters, the measurement of examinee and rater facets, and longitudinal usage of each category of the four tasks all point to the conclusion that the scoring of PRETCO-Oral is diachronically reliable. It should also be pointed out that in light of the narrow range of Infit (0.7–1.3), ratings of five raters, consisting of four new raters and one experienced rater, fall outside of the range.This suggests that more training and monitoring are essential on the part of new raters to warrant their rating quality, and experienced raters are not exempt from rater training. This finding seems to be at odds with that of Kim (2015) who maintained that all experienced raters' ratings exhibited a stable tendency. The poor rating quality of the experienced rater might be attributed to a number of factors, such as the varying degree of the difficulty of “parallel papers”, or the fatigue of raters, etc. Despite the intense rater training, raters still display somewhat differences concerning severity, which resonated with that of Lumley and McNamara (1995). This might be taken as the measurement error in educational testing.

The statistics of raters' usage of each category of the four tasks illustrate that excessive usage of middle rating categories of Reading Aloud is indicative of central tendency, making it difficult to discriminate examinees' speaking proficiency, and the highest level of rating category is seldom utilized in the rating of Interpretation and Presentation, which might be attributable to two factors, namely raters' misunderstanding of the rating category, or the problematic description of the scale. For the rating category with less than ten observations, several remedies were tailored to improve the scale, for instance, rewriting the descriptor of that category, combining it with the adjacent category, or simply omitting the category (Linacre, 2002a).

It is worth mentioning that raters' ratings might not strictly follow the pattern of the rating scale. Take the Interpretation rating scale, for example, five interviewees (Chris, Eric, Dora, Hanna, and Lucas) stated test of Interpretation involves the integrative ability to use English, and three of them (Chris, Eric, and Lucas) claimed to choose global scoring instead of counting how many sentences were completed by test-takers as is required in the rating scale of Interpretation. It turned out that their ratings fitted the Rasch model well, which may shed some light on the rater training or the modification of the rating scale of PRETCO-Oral.

**RQ 2**: To what extent can the construct of PRETCO-Oral be interpreted based on test scores?

The statistics using CFA based on scores of PRETCO-Oral prove that the construct of this speaking test was of high validity and the four tasks of Reading Aloud, Question and Answer, Interpretation and Presentation explained 43.6, 40.9, 75.7, and 75.7% variance of PRETCO-Oral on average respectively. The findings also resonated with data from the interview of raters. There are 30 references analyzed via NVivo 11 relating to and attesting to the overall construct validity of PRETCO-Oral. Viewpoints of raters interviewed, however, were divided upon the interpretation of the construct. One interesting thing is that teaching background may play a role in raters' perception upon conceptualizing PRETCO-Oral. Raters, like Hanna and Lily, also English major teachers, asserted that Practical English, by the name of PRETCO-Oral, refers to the application of English in people's daily communication, and this definition of construct seems to be ability focused (Bachman, 2007); Other six raters, also non-English major teachers, agreed that Practical English lies in applying English in the specific workplace, as is reflected in the tasks of PRETCO-Oral where the topics of Reading Aloud, Questions and Answer, etc., touch upon the introduction of a company, products or business agenda and so forth. This definition seems to be task-focused (Bachman, 2007). In this sense, raters' understanding of the construct of PRETCO-Oral blurred, echoing the statement of Bachman (2007) that neither the ability-focused nor the task-focused approach addresses the dilemma of discriminating language abilities from the contexts in and of itself (Cai, 2020).

Compared to the findings of quantitative analysis about Reading Aloud and Question and Answer, several raters also questioned their validity similarly. Eric, Lucas, and Shelly pointed out that Reading Aloud pertains to reading comprehension instead of speaking ability, as is evidenced by Prior et al. (2011) who substantiated that reading aloud made no difference to silent reading for high-grade students. There is even no need to involve Reading Aloud in PRETCO-Oral according to Rater Chad.

*I score PRETCO-Oral in a reversed order, and test-taker's pronunciation and intonation can be judged based on his performance on tasks of Presentation, Interpretation etc. – Chad*.

Question and Answer equal fast reading in that this task calls for scanning for specific information relevant to “Questions” and “Answers”, according to Chris, Lily, and Lucas. Chad even implied that Reading Aloud is unnecessary because the rating of examinees' pronunciation, intonation, and stress could be accomplished in light of their performance on the other three tasks. Additionally, the test format of Question and Answer is semi-direct which might fail to predict people's ability to communicate in a real workplace (Qian, 2009) although half of the interviewers took a positive view of the potential of this task to measure test-takers' communicative ability.

For the two tasks of Interpretation and Presentation, raters presented their consent that the former examines students' oral Interpretation ability in daily foreign communication and foreign business, and the latter measures test-takers' ability to communicate coherently in English, as is required in the syllabus of PRETCO-Oral. To put it differently, the two tasks assess examinees' comprehensive ability to use English in daily communication and workplace. It's worth noting, however, that there are some criticisms from Shelly and Chris. Shelly conceives of the two tasks as measuring the same thing, which is similar to oral writing. The difference lies in whether there is a Chinese prompt or not. And Chris criticizes the inauthenticity of the Presentation task that lacks an authentic scenario.

**RQ 3**: Does the construct of PRETCO-Oral keep the same manner longitudinally?

Longitudinally, the separate CFA models of four occasions fit the data well as a whole, which suggests an overall steady construct of PRETCO-Oral, except for the second occasion where the value of the index of RMSEA was considered to be too large. The test of measurement of invariance of PRETCO-Oral across the four sessions of assessments indicates that metric invariance is supported while scalar and residual non-invariance are also found, which means that the measurement of PRETCO-Oral might not be fully equitable diachronically. Several factors should be held accountable, including varying levels of English proficiency of test-takers for each occasion, different degrees of test difficulty, subjective raters' ratings, and so forth.

Drawing on the interview data, it can be seen that although eight raters reached a consensus that the test maintained its validity across the four occasions, most raters assumed that the parallel sheets on each occasion also kept equitable. There are two raters, Eric and Dora, who were engaged in rating examinees' responses from two “parallel” sheets and sensed evident differences with regard to task difficulty. Taking Reading Aloud, for instance, we calculated the readability of four “parallel” reading passages (55.8 “slightly difficult”, 55.2 “slightly difficult”, 73.3 “slightly easy”, 60.6 “standard”) and found that there exists a difference of difficulty between two extremes.

**RQ 4**: To what extent can the construct of PRETCO-Oral be interpreted from the perspective of PRETCO-Oral test-takers?

The statistic using CFA based on questionnaire data displays that the model and data fit well, which serves as important evidence for the construct of PRETCO-Oral. The four tasks of Reading Aloud, Question and Answer, Interpretation, and Presentation accounted for 34.8, 51.8, 82.8, and 74.0% variance of PRETCO-Oral from the test-takers' perspective, which seems identical to the findings resulting from the analysis of PRETCO-Oral scores.

It might be safe to say that examinees' perception of the rating scale contributes a great deal to the interpretation of the construct of PRETCO-Oral. The factor loadings of Reading Aloud and Question and Answer, however, were much lower than those of Interpretation and Presentation, which may be due primarily to the inefficiency of Reading Aloud in discriminating students' speaking proficiency as a result of the central tendency of raters' rating, or test-takers' unfamiliarity of Questions and Answer in spite of the 20 min of training before sitting for the test. It was felt that test-takers were not familiar with how to initiate questions or answer questions in relation to the prompt, according to four interviewees, namely, Chris, Eric, Dora, and Lucas. Examinees' unfamiliarity with the task may act as a construct irrelevant variable that jeopardizes the validity of PRETCO-Oral.

*… I really doubt whether students could understand the requirement of this test item… – Chris*.

*… Time was quite limited, and the test item was changed to another one before test-takers could react. Some students saw, for example, some of the underlined parts in the question, but they did not know that they were going to initiate questions about the underlined parts… – Eric*.

*… it is estimated that this test may fail to examine the intended ability, because students may not grasp how to deal with this task … – Dora*.

*… for a number of test-takers, it was found that there was no fluctuation in the audio wave, which means that they probably do not know what to do. – Lucas*.

## Conclusion

This study explored the construct of PRETCO-Oral by dint of longitudinal Rasch model, CFA, and interview directed at corroborating the interpretation of PRETCO-Oral. For the rating reliability, though there exist some significant differences between raters' severity across the four occasions of tests and the ratings of only one or two raters slightly misfit or overfit the Rasch model in each occasion referring to the narrow range of Infit, the overwhelming majority of raters were able to distinguish examinees' speaking proficiency and maintain the high level of intra-rater consistency in each occasion, indicating a high level of reliability of the overall scoring of PRETCO-Oral longitudinally. For the construct of PRETCO-Oral, the two hypothetical models fit the data of PRETCO-Oral scores and those from the questionnaire, lending support to the finding that the variance of PRETCO-Oral could be explained by the four tasks longitudinally. Meanwhile, test-takers' perception of the construct of PRETCO-Oral also confirms the construct validity of the test. As a whole, this study has addressed one central problem, i.e., longitudinal evidence for the construct of PRETCO-Oral and some evidence from the test-takers has also been collected to validate the speaking test.

Some implications for the improvement of the test have also been found on the issue of construct irrelevance and construct underrepresentation. These threats include inauthenticity of task of Question and Answer and Presentation, unsatisfactory validity of Reading Aloud, and test-takers' unfamiliarity with the task of Question and Answer, deficiency of rating scales of Reading Aloud and Presentation. Further studies and corresponding modifications can be done to enhance the validity of the speaking in the future. For example, experienced rater teams can be formed and new tasks can be designed to measure students' speaking ability. The limitations of the current study pertain to the deep interpretation of test-takers' and raters' perceptions of the construct of PRETCO-Oral. Further studies can probe into the cognitive process of test-takers by means of verbal protocol or adopt eye tracking to understand the raters' behaviors. As the constraint of practicality, the feedback from test-takers is collected only once. Further studies can collect more information to depict a more comprehensive picture longitudinally.

## Data Availability Statement

The raw data supporting the conclusions of this article will be made available by the authors, without undue reservation.

## Ethics Statement

Ethical review and approval was not required for the study on human participants in accordance with the local legislation and institutional requirements. The patients/participants provided their written informed consent to participate in this study. Written informed consent was obtained from the individual(s) for the publication of any potentially identifiable images or data included in this article.

## Author Contributions

ZY was responsible for test data collection and paper-writing. YZ examed the whole paper and grouped the team works. ZLi transcribed and analyzed the qualitative data by Nvivo and did the formatting. ZLin took the final exam revision and saw for further data analysis and confirmation. All authors contributed to the article and approved the submitted version.

## Funding

This work was supported by the Graduate Research and Innovation Projects of Guangdong University of Foreign Studies [21GWCXXM-070].

## Conflict of Interest

The authors declare that the research was conducted in the absence of any commercial or financial relationships that could be construed as a potential conflict of interest.

## Publisher's Note

All claims expressed in this article are solely those of the authors and do not necessarily represent those of their affiliated organizations, or those of the publisher, the editors and the reviewers. Any product that may be evaluated in this article, or claim that may be made by its manufacturer, is not guaranteed or endorsed by the publisher.
