# Supplementary material for: Interrogating the Construct of PRETCO-Oral: Longitudinal Evidence From Raters and Test-Takers
Source: Front Psychol. 2022 Jul 12;13:896453. doi: 10.3389/fpsyg.2022.896453 (PMC9322943; doi:10.3389/fpsyg.2022.896453)
Supplement: Supplementary file 1 [file Presentation_1.pdf]

Supplementary information

Appendix A Test Sample

Part 1 Reading Aloud

In this part, there is a short speech shown on the screen for you to read aloud.

Task:

- First, you'll have 1 minute to read the speech silently for preparation.
- Then, please read aloud the speech once within 1.5 minutes.

Good morning everybody. Welcome to our annual sales conference. It's great to see so many of you -- old faces and new ones! Now we're going to have two very busy days as usual, but I am sure you'll enjoy them. As soon as I finish, we'll begin with our first session. Our sales managers will give their regional sales reports. From their reports, we'll see the overall picture of our sales last year.

Tomorrow morning we'll discuss ways of marketing the new products to our potential clients. This will be an intensive session. And that will bring us to the end of the conference and a dinner party. I wish you all an enjoyable and successful time.

Thank you!

Part 2 Questions & Answers

In this part, you are required to ask three questions and then answer three questions according to what is shown on the screen.

TASK ONE:

- Suppose you want to participate in the event.
- You'll have 1 minute to read through the poster SILENTLY.
- Then, you are required to ask 3 questions for detailed information.
- You'll have 10 seconds to finish each question.

goodfridayappeal

To bring together the community
To raise money for the Royal Children's Hospital

Date: April 3rd, 2015
Time: 8 a.m. – Midnight
Place: 132 Community Center

Ways to get involved:
*Make a donation ONLINE
*Call 1300 APPEAL: 1300 277325

Official volunteer groups:
*Uncle Bobs Club – Phone: 9335 6300
*RCH Society – Phone: 9848 9006

goodfridayappeal

To bring together the community
To raise money for the Royal Children's Hospital

Date: Question 1
Time: 8 a.m. – Midnight
Place: Question 2

Ways to get involved:
*Make a donation ONLINE
*Call 1300 APPEAL: 1300 277325

Official volunteer groups:
*Uncle Bobs Club – Phone: 9335 6300
*RCH Society – Phone: Question 3

Question 1:

Part 3 Chinese-English Interpretation

In this part, you're required to translate a short speech into English orally.

Task:

- First, you'll have 1 minute to prepare.
- Then, you should begin to do the translation when hearing a signal sound.
- You'll have 2 minutes to finish your translation.

我们是一家生产玩具的大公司，在世界各地都有分公司。每年都有许多大学毕业生来我们公司工作。我们欢迎更多的大学毕业生加入我们公司。如果你有意申请我们公司的职位，你可以访问我们公司的网站，查询详细的信息。

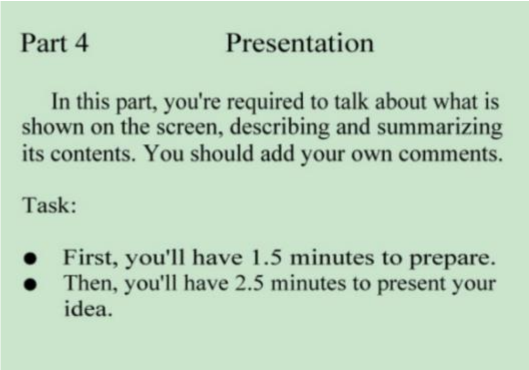

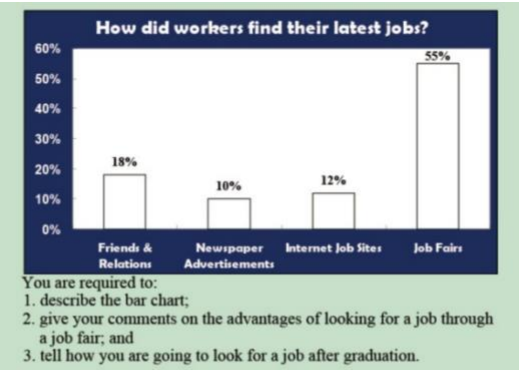

Appendix B Questionnaire

(1 = Strongly Disagree 2 = Somewhat Disagree 3 = Average 4 = Somewhat Agree 5 = Strongly Agree)

Reading Aloud

I can understand the meaning of the passage when reading.	1 2 3 4 5
I can pay attention to my pronunciation during my reading.	1 2 3 4 5
I can pay attention to my intonation during my reading.	1 2 3 4 5
I can focus on my fluency when reading	1 2 3 4 5
I can pay attention to how to break sentences when reading.	1 2 3 4 5

Question and Answer

I know how to ask and answer questions upon hearing the instructions.	1 2 3 4 5
I can get familiar with the topic of the test when taking the test.	1 2 3 4 5
I can answer questions assuming there is an audience.	1 2 3 4 5
I can pay attention to the content and words when answering questions.	1 2 3 4 5
I can be aware of the scenario and role engaged when answering questions.	1 2 3 4 5

Interpretation

I can choose the right words or expressions when I interpret.	1 2 3 4 5
I interpret consistently in tone, sentence, and content.	1 2 3 4 5
I pay attention to the appropriacy of the content when I interpret.	1 2 3 4 5
I make sure I break the sentences properly when I interpret.	1 2 3 4 5
I pay attention to achieving communicative effect when I interpret.	1 2 3 4 5

Presentation

I can choose the right words when I make the presentation.	1 2 3 4 5
I can express myself in correct sentences.	1 2 3 4 5
I can describe all the charts properly.	1 2 3 4 5
I can organize the appropriate language make a comment.	1 2 3 4 5
I can focus on the coherence of my presentation.	1 2 3 4 5

Appendix C Rating Criteria of PRETCO-Oral

Reading Aloud

Score	Pronunciation and Intonation	Fluency
4	Correct pronunciation and intonation, proper stress, pause and dividing of meaning groups	The reading is fluent and clear.
3.5	Basically correct pronunciation and intonation, basically appropriate stress, pause and dividing of meaning groups	The reading is basically fluent and clear.
3	There are a few pronunciation and intonation errors, but they do not affect the understanding of audiences.	The reading is basically smooth.
2.5	There are more pronunciation and intonation errors, but audiences could basically understand the reading.	Sometimes there are pauses and repetitions in reading.
2	There are many errors in pronunciation and intonation, which affect the understanding of reading.	The reading is not very smooth, and there are often pauses and repetitions.
1	Reading aloud is basically incomprehensible	It's difficult to read aloud.
0	Can or do not read.	

Question and Answer

Score	Content and Expression	Language
4	Can finish all questions and answers, the content is complete and relevant, and the expression is basically clear and coherent.	The statement is in line with the English norm.
3.5	Can finish 5 questions and answers, the content is relevant and the expression is basically correct.	The statement is basically in line with the English norm.
3	Can finish 4 questions and answers, the content is basically complete and relevant with occasional pauses, but basically coherent.	The statement slightly accords with the English norm; sentences are basically correct and words are basically appropriate.
2.5	Can finish 3 questions and answers, the content is basically complete and relevant, with occasional pauses, but basically coherent.	Sentences are basically correct and words are basically appropriate.
2	Can finish 2 questions and answers, the content is incomplete, there are often pauses or repetitions, barely understandable	There are many mistakes in the statement.
1	Can barely answer 1 question.	There are many mistakes in the statement, which is difficult to understand.
0	Not answering the question	

Interpretation

Score	Content and Expression	Language
4	Can translate all the sentences and express the original meaning correctly.	The statement is in line with the English norm.
3.5	Can complete the interpretation of four sentences, basically correctly express the meaning of the original text, and only omit a few secondary information.	The statement is basically in line with the English norm.
3	Can basically complete the interpretation of four sentences.	The statement slightly accords with the English norm; sentences are basically correct and words are basically appropriate.
2.5	Can complete the translation of three sentences and basically express the main meaning of the original text.	Sentences are basically correct and words are basically appropriate.
2	Can complete the translation of two sentences and basically express the main meaning of the original text.	There are many mistakes in the statement.
1	Can only complete the translation of one sentence segment and basically express the main information of the original text.	There are many mistakes in the statement, which is difficult to understand.
0	No answers or all wrong answers	

Presentation

Score	Content and Expression	Language
4	Can clearly and coherently introduce the important information contained in the picture with comments	The statement is in line with the English norm.
3.5	Can introduce the important information contained in the picture clearly and coherently with comments	The statement is basically in line with the English norm.
3	Can basically convey the important information contained in the picture coherently.	The statement slightly accords with the English norm; sentences are basically correct and words are basically appropriate.
2.5	Can express the important information of the picture with a small amount of pause or repetition	Sentences are basically correct and words are basically appropriate.
2	Can barely express the main information of the picture, but it has many omissions and often pauses or repeats.	There are many mistakes in the statement, some of which is difficult to understand.
1	Basically cannot express the picture information, the narration is confused, and only one or two sentences can be understood.	There are many mistakes in the statement, which is difficult to understand.
0	There is no answer or the answer cannot be understood	
